# Whole genome sequencing of an ethnic Pathan (Pakhtun) from the north-west of Pakistan

**DOI:** 10.1186/s12864-015-1290-1

**Published:** 2015-03-12

**Authors:** Muhammad Ilyas, Jong-Soo Kim, Jesse Cooper, Young-Ah Shin, Hak-Min Kim, Yun Sung Cho, Seungwoo Hwang, Hyunho Kim, Jaewoo Moon, Oksung Chung, JeHoon Jun, Achal Rastogi, Sanghoon Song, Junsu Ko, Andrea Manica, Ziaur Rahman, Tayyab Husnain, Jong Bhak

**Affiliations:** National Centre of Excellence in Molecular Biology, University of the Punjab, Lahore, Pakistan; Personal Genomics Institute, Genome Research Foundation, Suwon, Republic of Korea; Theragen Bio Institute, TheragenEtex, Suwon, Republic of Korea; The Genomics Institute, Biomedical Engineering Department, UNIST, Ulsan, Republic of Korea; Korean Bioinformation Center, Korea Research Institute of Bioscience and Biotechnology, Daejeon, Republic of Korea; Department of Zoology, University of Cambridge, Downing Street, Cambridge, CB2 3EJ UK

## Abstract

**Background:**

Pakistan covers a key geographic area in human history, being both part of the Indus River region that acted as one of the cradles of civilization and as a link between Western Eurasia and Eastern Asia. This region is inhabited by a number of distinct ethnic groups, the largest being the Punjabi, Pathan (Pakhtuns), Sindhi, and Baloch.

**Results:**

We analyzed the first ethnic male Pathan genome by sequencing it to 29.7-fold coverage using the Illumina HiSeq2000 platform. A total of 3.8 million single nucleotide variations (SNVs) and 0.5 million small indels were identified by comparing with the human reference genome. Among the SNVs, 129,441 were novel, and 10,315 nonsynonymous SNVs were found in 5,344 genes. SNVs were annotated for health consequences and high risk diseases, as well as possible influences on drug efficacy. We confirmed that the Pathan genome presented here is representative of this ethnic group by comparing it to a panel of Central Asians from the HGDP-CEPH panels typed for ~650 k SNPs. The mtDNA (H2) and Y haplogroup (L1) of this individual were also typical of his geographic region of origin. Finally, we reconstruct the demographic history by PSMC, which highlights a recent increase in effective population size compatible with admixture between European and Asian lineages expected in this geographic region.

**Conclusions:**

We present a whole-genome sequence and analyses of an ethnic Pathan from the north-west province of Pakistan. It is a useful resource to understand genetic variation and human migration across the whole Asian continent.

**Electronic supplementary material:**

The online version of this article (doi:10.1186/s12864-015-1290-1) contains supplementary material, which is available to authorized users.

## Background

Sequencing technology is improving fast, with a drastic reduction of its costs [1]. These rapid advances have greatly expanded our understanding of human genetic diversity and population history [2], enabling us to investigate variants with health consequences and paving the way to personalized medicine [3]. Genome wide association studies (GWAS) have characterized the function of thousands of common SNVs, but there are still millions of variants left unexplored [4]. Therefore, whole genome sequencing is necessary for a detailed study of rare genomic variants. A number of international consortia have started sequencing the whole genomes of large panels, including the 1000 Genomes Project (www.1000genomes.org), the Personal Genome Project (www.personalgenomes.org), and the 100 Malay genomes [5]. These consortia, as well as several geographically more restricted projects, aim to understand the functional aspects of both common and unique variants in humans. In the future, we can expect all distinct ethnic groups to have their genomes sequenced.

Pakistan lies at a junction of the Indian sub-continent in the East, the Central Asian States in the West, and China towards its North. It has a unique socio-religious-cultural history, in addition to a number of ethnic and linguistic groups such as Punjabi, Pathan (Pakhtuns), Sindhi, and Baloch (Additional file 1: Figure S1) [6]. While a number of these groups have been included in genetic panels typing microsatellites and SNPs [7], only one male Pakistani individual of unknown ethnic origin has been sequenced so far (Additional file 1: Figure S2) [8]. Here we report the first whole-genome sequence and analysis of a Pathan male (Pakistani national). Genomic variations including single nucleotide variations (SNVs), small insertions and deletions (indels), and copy number variation regions (CNVRs) were identified by aligning the Pathan genome sequence to the Human Reference Genome (hg19). Variants were then annotated and scanned for associated functions along with SNVs that could modulate drug response. Possible deleterious non-synonymous SNVs (nsSNVs) were investigated for potential effect on the pharmacokinetics and pharmacodynamics of drugs. Additionally, multiple analytical approaches were used to assess the influence of ancestral contributions within the Pathan (PTN) genome.

## Results and discussion

### Genome sequencing and variants identification

DNA extracted from blood was sequenced with paired-end reads of 90 bp using the Illumina HiSeq2000 sequencer, producing 1,069,127,687 reads. A total of 83.3 Gb of sequences were generated and aligned to the human reference genome (without Ns, 2,861,343,702 bp), covering 98.2% of the reference genome at an average 28.5× depth (Additional file 2: Table S1).

We identified a total of 3,813,440 SNVs, of which 3,683,999 (96.6%) were reported in the dbSNP database [9] and 129,441 were novel (Table 1) which were further compared with the novel variants count of other individual genomes from literature (Additional file 1: Figure S3) [10-19]. There were 1,272,912 homozygous and 2,540,528 heterozygous SNVs. A total of 18,547 SNVs were found in coding DNA sequence (CDS) regions, 25,481 in 3’ untranslated regions (UTR), and 4,969 in 5’ UTRs. A total of 10,315 SNVs in 5,344 genes were non-synonymous (nsSNVs).

**Table 1:** **Summary of SNVs found in Pathan’s genome and overlaps with dbSNP137**

**Total**	**Homozygous**	**Heterozygous**	**SNVs mapped to dbSNP (v137)**	**% of SNVs mapped to dbSNP**	**Novel**	**% of Novel**
**SNVs**	**SNVs**	**SNVs**			**SNVs**	**SNVs**
3,813,440	1,272,912	2,540,528	3,683,999	96.6%	129,441	3.39%

**Figure 1:**
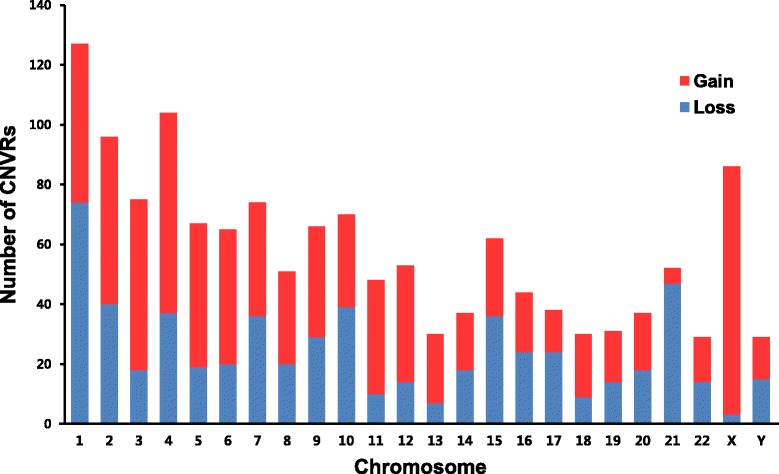
**Copy number variation regions in Pathan genome.** Copy number variations counts distributed in each chromosome.

A total of 504,276 short indels (up to ±20 bases) were observed, of which 306,128 were found in intergenic regions, 237 in CDS regions, and 193,308 in intron regions. Additionally, 1,503 CNVRs were found, 713 of which were classed as duplicated and 790 as deleted, affecting 2,364 overlapped genes (Additional file 3: Table S2). A total of 65 CNVRs had not previously been described in the database of genomic variants (DGV; http://projects.tcag.ca/variation/). Figure 1 shows the number of gained and lost CNVRs in each chromosome. ANNOVAR was used for detailed annotation analysis of CNVRs to identify genes associated with these regions (Additional file 4: Table S3).

### Functional classification and clinical relevance of variants

All 10,315 nsSNVs found in the Pathan genome were further scrutinized for their possible functional effects using computational prediction methods (SIFT and Polyphen2), resulting in 43 nsSNVs in 43 genes being classified as functionally damaging (Additional file 5: Table S4). Additionally, nsSNVs were annotated using ClinVar for their clinical relevance, and we found that 31 coding SNVs are associated with several diseases (Additional file 6: Table S5). Of particular note are an SNV (rs1049296, Pro570Ser) in the *TF* gene [20], which affects Alzheimer’s susceptibility; Ser217Leu in *ELAC2* gene (rs4792311), which is implicated in genetic susceptibility to hereditary prostate cancer [21]. The rate of prostate cancer is low in Pakistan (3.8%) [22], as compare to Americans and Caucasian [23]. Three coding SNVs on *GHRLOS* (rs696217, Leu72Met), *SERPINE1* (rs6092, Ala15Thr), and *PPARG* (rs1801282, Pro12Ala) which all have links with obesity [24-26]. About 22.2% of Pakistanis are reported to be obese which is close to European (~24%) and United States populations (~19%) [27-29].

We also found three pathogenic SNVs in genes associated with hair, skin and pigmentation: *EDAR* (rs3827760, Val370Ala), *SLC45A2* (rs16891982, Phe374Leu), and *TYR* (rs1042602, Ser192Tyr) [30-32]. In addition, we detected a SNV (rs17822931, Gly180Arg) in *ABCC11*, which is responsible for wet earwax which was also found in the Pakistani PK1 genome [33].

One of the variants (rs1065852, Pro34Ser) in the *CYP2D6* gene is responsible for poor metabolism of debrisoquine, an adrenergic-blocking medication used for the treatment of hypertension [34]. Also, two SNVs in the *TPMT* (rs1142345, Tyr240Cys and rs1800460, Ala154Thr) are known to have a pathogenic effect and lead to thiopurine methyltransferase (TPMT) deficiency [35,36]. Moreover two nsSNVs (rs2056899 and rs140980900) of *CYP4A22* and *GGT5* genes in the Arachidonic acid metabolism pathway were found (Additional file 7: Table S6). Arachidonic acid in the human body usually comes from dietary animal sources, such as meat, eggs, and dairy. Meat is an important part of a Pathan’s diet, usually consumed at least once a day, often in the form of kabab (minced meat fried in oil), or curry [37].

Comparative genomic analysis was done using Pathan (PTN) genome and the other previously published Pakistani (PK1) genome. Non-synonymous variants from Pakistani (PK1) genome were annotated for investigating associated diseases. Out of ~8,000 nsSNVs only 37 variants (three novel) were found linked with certain disorders. Eight clinically relevant SNVs were detected overlapped with Pathan (PTN) genome. We found no damaged variants responsible for Alzheimer’s, obesity and heart related diseases just like we found in Pathan (PTN) genome. An SNV (rs1057910; *CYP2C9*) was observed in PK1 genome which is known for Wafarin response. Moreover, a pathogenic mutation (rs1169305) was seen in the *HNF1A* gene which may become a cause of diabetes in the PK1 individual.

Most of the clinically relevant variants adopted in this study were originally described in Caucasian populations. While this result might be a consequence of the genomic affinities of the Pathan genome with other Caucasian populations, it might also reflect a bias due to most of the GWAS work being carried out on Caucasian populations [38]. Therefore a cohort study in the Pakistani population will be required for authentication.

### Pharmacogenomics analysis

Damaging nsSNVs were annotated using PharmGKB and DrugBank databases [39,40]. A large number of variants were associated with susceptibility to poisonous drugs, while others nsSNV were linked to the efficacy of medicines used in the treatment of diseases such as depression, diabetes mellitus, Alzheimer disease, arthritis and so on (Additional file 8: Table S7). After discovering the possibly damaged variants found in SIFT and Polyphen2, the consensus of both datasets was further analyzed in order to find the most probable impact of these deleterious variants in terms of drug targeting, transport, and metabolism. We found nsSNVs that affect the function of drugs (two transport, five enzymatic, and four drug targets). A variant rs1801133 (A222V in MTHFR gene) was found associated with increased risk of metabolic syndrome when treated with antipsychotics [41]. Our donor has high chance of having decreased diastolic blood pressure if treated with benazepril [42]. One of the variants (rs1799930, R197Q in *NAT2* gene) was associated with increased risk of toxic liver disease when treated with ethambutol, isoniazid, pyrazinamide, and rifampin [43]. We also observed an SNV (rs1065852, Chr22:42526694 G > A) which made this individual use escitalopram for depression and other anxiety [44]. The detail list of those drugs can be found in Additional file 9: Table S8.

### Comparison with other Pathan individuals

**Figure 2:**
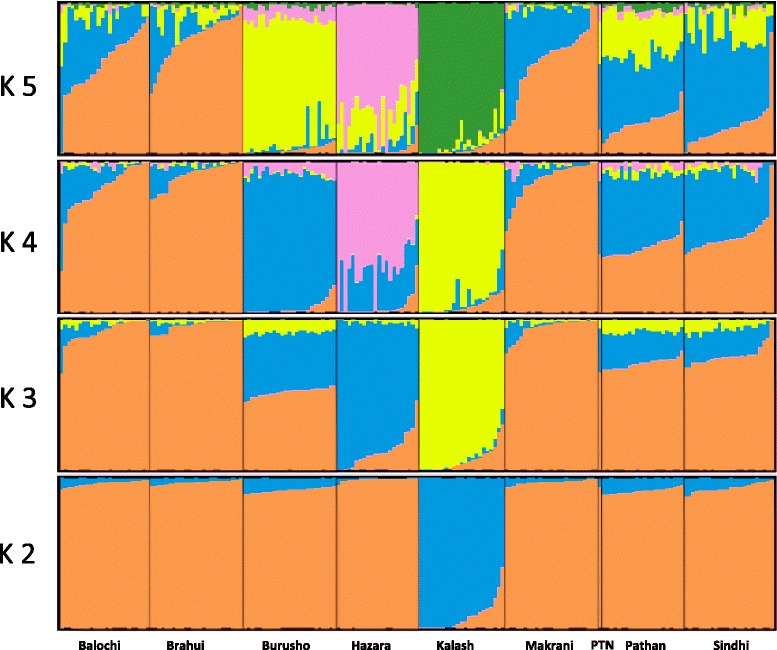
**Admixture results of Pathan (PTN) individual to other ethnic groups in South Asia.** Admixture results for K = 2 and K = 5 for the Pathan individual combined with eight ethnic genomes from HGDP dataset. The analysis was based on 643,281 SNVs. Each individual is represented by a vertical line, divided into colored segments that represent membership coefficients in the subgroups.

We investigated how representative our Pathan genome was of that ethnic group by comparing it to another twenty-two Pathan individuals in the HGDP-CEPH panel [7], which had been typed for ~650 k SNVs, together with a further 190 individuals from another eight South Asian (Pakistani) populations from the same panel. Admixture analysis was performed based on 643,281 SNVs (thinned to avoid LD). We considered the cluster membership from STRUCTURE (from K = 2 to K = 5), the Pathan (PTN) genome composition was within the variability observed within the Pathan sample from the HGDP (Figure 2). Similarly, in a multi-dimensional scaling (MDS) plot, the Pathan genome fell within the other Pathan individuals (Additional file 1: Figure S4). Taken together, these two results confirm that the Pathan genome presented in this paper is representative of the Pathan ethnic group. These results are also in line with the self-reported ancestry of the subject, with all his grandparents coming from Afghanistan to Khyber Pakhtunkhwa (Pakistan).

### mtDNA and Y-chromosome analyses

The full mitochondrial genome of the Pathan individual was generated by mapping its reads to the revised Cambridge reference sequence (rCRS) [45]. Adenine and thymine (AT) content of the genome was 55.5%, while guanine and cytosine (GC) content was 44.5%. A total of 57 SNVs were found in the Pathan mitochondrial genome, 13 of which had not been previously reported. The variants were then mapped with MitoVariome [46] to identify the mitochondrial haplogroup of our Pathan individual. A total of 14 SNVs were diagnostic of the H2 haplogroup (Additional file 10: Table S9), which has been argued to be of exclusive Caucasian origin, and its marginal occurrence in Pathans reflects admixture [47].

The AT and GC contents of the Y-chromosome were 39.87% and 60.13%, respectively. A total of 13,724 SNVs were identified, of which 4,423 were novel. The observed Y-chromosomal SNVs were annotated as markers for the L1 haplotype of clade L. Haplogroup L has high frequency in Pakistan (14%) as compare to India (6.3%), Turkey (~4%) and Caucasians (~6%) [48-50].

### Demographic history analysis

**Figure 3:**
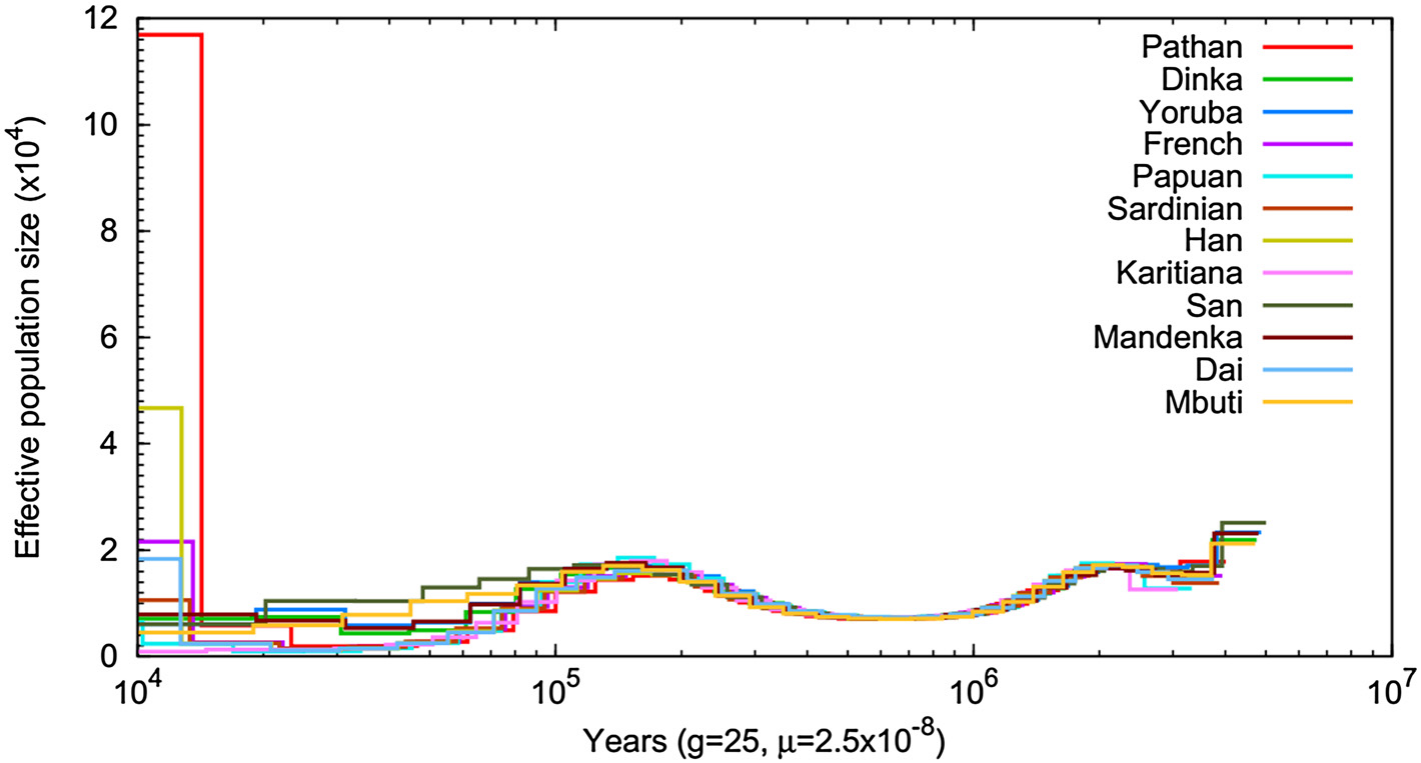
**Inferred historical population sizes by Pairwise Sequential Markovian Coalescent analysis.** PSMC (Pairwise Sequentially Markovian Coalescent) analysis was performed to reconstruct the demographic population history of Pathans, compared with a set of 11 HGDP genomes from around the world (Africa: Dinka, Yoruba, Mandenka, Mbuti, San; Asia: Dai, Han; Europe: French, Sardinian; Oceania: Papuan; Americas: Karitiana).

We inferred the demographic history of the Pathan using the pairwise sequentially Markovian coalescent (PSMC) model [51] (Figure 3), and compared it to a panel of worldwide populations based on a number of HGDP genomes [52]. As previously reported, all populations share a similar demographic history between 1 million to 200kyr ago. From 200kyr ago to 20kyr ago, the Pathan follow a similar trajectory to other Asian and European populations, with an inferred effective population size smaller than African populations, reflecting the out of Africa bottleneck. Over the last 20 k years, the Pathan shows an explosion in effective population size, contemporaneous to other Eurasian populations but much greater in magnitude. The very large effective population size likely reflects admixture between European and Asian lineages giving rise to modern Pathans (as also suggested by the analysis of mtDNA and Y-chromosome), rather than an actual increase in census sizes.

## Conclusions

Here we present, for the first time, the whole genome of a Pathan individual from a north-west province (Khyber Pakhtunkhwa) of Pakistan. Our analysis provides a detailed view of the Pathan genome diversity and functional classification of variants and its impact in pharmacogenomics. A large scale analysis of diverse genomes is needed to help researchers around the world in understanding genetic diversity and functional classification of variants along with pharmacogenomic traits and associated drugs that would be use as personalized medicine.

## Methods

### Subject selection and ethical statement

This study has been performed in accordance with Declaration of Helsinki and has been approved by the Institutional Review Board Genome Research Foundation (GRF) with IRB-REC-2011-10-003. Signed informed consents were obtained from the participant in this study and his family members' consent on publishing the entire content of the genome and phenotype information, as well as personal identifying information (such as age, sex and location).

There are documented cases of his family members with hypertension, heart problems, neuro disorders, diabetes and obesity. His father has been diagnosed for cardiovascular disorder, hypertension and Alzheimer’s. His mother has osteoarthritis and grandparents were died due to heart attack, cancer and hypertension.

### Data sources

The UCSC reference genome (hg19, February 2009), dbSNP version 137 and genome annotations, were downloaded from the database (www.genome.ucsc.edu). Genomes from HGDP-CEPH panel of 190 individuals belong to eight South Asian (Balochi, Brahui, Burusho, Hazara, Kalash, Makrani, Pathan and Sindhi) populations, which had been typed for ~650 k SNVs were retrieved from the publically available database.

### DNA extraction

Genomic DNA was extracted from the arterial blood lymphocytes of a Pakistani Pathan thirty-year-old male residing in the North-West province of Pakistan. QIAamp DNA Blood Mini Kit was used for DNA extraction from the blood (Qiagen). Tecan’s Infinite F200 nanodrop was used to assess DNA purity, 1.7 % agarose gel electrophoresis to confirm DNA size (presence of high molecular weight DNA) and Invitrogen’s Qubit fluorometer to determine the DNA concentration.

### Cytogenetic analysis

Karyotyping was carried out with cultured peripheral blood lymphocytes using standard techniques, and GTG banding was used to identify chromosomal aberrations, which is useful for identifying genetic diseases through the photographic representation of the entire chromosome complement [53]. No obvious chromosomal abnormalities were found in the cytogenetic analysis through G-banded karyotyping chromosome imaging (Additional file 1: Figure S5).

### Library preparation and whole genome sequencing

Two paired-end libraries were prepared from 1.1 μg of gDNA using Illumina TruSeq DNA Preparation Kit, following Illumina’s standard protocol (Paired-end Library Preparation Kit, Illumina, SanDiego, CA, USA). Shearing of gDNA was done using Covaris S series (Covaris, MS, USA). Following end repair, A-tailing, and adaptor ligation, DNA in the 500–600 bp range was purified from a 2% agarose gel. DNA was then PCR enriched for a total of ten cycles. Proper DNA size was then confirmed with the Agilent Bioanalyzer, followed by qPCR quantification with Roche Light Cycler 480 II and Kapa Biosystems reagents.

Cluster generation was performed on an Illumina cBot and the libraries were sequenced on an Illumina HiSeq 2000 following the Paired-End protocol. Sequences can be accessed at NCBI SRA, with accession number SRA092047. The rest of our analysis was initiated from the FASTQ files provided by Illumina's downstream analysis CASAVA software suite.

### Mapping and alignment to the reference genome

The genome sequences were aligned with the human reference genome (hg19) using Burrows-Wheeler Aligner (BWA; version 0.5.9) [54] and SAMtools 0.1.16 [55] with the default options, except “aln -t 3 -l 45 -k 2” options. Alignment files were then merged into a single BAM file, marked for duplicates using Picard 1.59 (http://picard.sourceforge.net) and base quality scores were recalibrated using Genome Analysis Toolkit (GATK v1.4) [56].

### SNVs, short indels, and CNVs calling

SNVs and small indels ranging from 1 to 20 bases were identified using Genome Analysis Toolkit (GATK v1.4) with HARD_TO_VALIDATE: MQ0 ≥ 4 and ((MQ0/(1.0 × DP)) > 0.1), 2) QualFilter = QUAL < 10, 3) DepthFilter = DP < 5 or DP < 200 options. All SNVs were further characterized based on zygosity predictions from BWA consensus model. ReadDepth 0.9.7 was used for identification of copy number variations with bin size 0.01 [57]. Copy number calls smaller than 1.3 were taken as loss and greater than 2.6 as gains. The minimum size taken was 1,000 bp. All the found variants were then further annotated in OMIM (www.omim.org), DGV (http://projects.tcag.ca/variation/), SIFT [58], PolyPhen2 [59], and ClinVar [60] using ANNOVAR [61].

Results from SIFT and PolyPhen2 were compared and common possibly damaged variants were retrieved. Nonsynonymous SNVs with functional abnormality from Pathan genome were then annotated against publically available datasets like DrugBank and PharmGKB to investigate association with drugs involved in different activities which includes the list of genes/variants involved in drug transport, metabolism and drug targets [39,40]. The methodology used for pharmacogenomic analysis has been previously reported [62].

### Multidimensional scaling and admixture analysis

To test the representativeness of the Pathan (PTN) genome, we compared it other Pathan individuals that had been typed for ~650 k SNPs in the HGDP-CEPH panel, together with individuals from another eight Central Asian populations. We use multi-dimensional scaling to visualize the relationships among all this individuals, using 643,281 SNVs thinned using PLINK (50 basepair sliding windows, advancing in steps of 10, removing any SNV with R^2^ bigger than 0.1 with any other SNV within the same window). MDS components were obtained using the PLINK mds-plot option based on the identity-by-state (IBS) distance matrix. Admixture analysis was performed using the program STRUCTURE to identify the presence of diverse ancestral relation of the Pathan (PTN) genome with others [63]. We explored values of K from 2 to 5, and chose the K value that gave the lowest cross-validation error.

### Pairwise sequentially markovian coalescent analysis

We conducted a PSMC (Pairwise Sequentially Markovian Coalescent) analysis to reconstruct the demographic population history of Pathans [51]. We compared the Pathan genome to a set of 11 HGDP genomes from around the world (as published by Meyer *et al.*) [52]. We first used samtools to extract the diploid genomes from their BAM files aligned to hg19, and excluded sex chromosomes and mitochondrial genomes because they are haploid. In PSMC, we used the command line options -N25 -t15 -r5 -p "4 + 25*2 + 4 + 6" that have been successfully used in previous similar analyses of human and great apes [64].

## Additional files

Additional file 1: Figure S1. (Map of South Asia showing the Pathan/Pakhtun ethnic group in Pakistan and Afghanistan). **Figure S2** (Comparative variant count of other reported individual genomes with Pathan genome. **Figure S3** (Novel SNVs in personal genomes in thirteen different ethnic groups). **Figure S4** (Comparing Pathan ethnic genome with other twelve diverse ethnic genomes from South Asia). **Figure S5** (Cytogenetic analysis through GTG banding karyotype) and legends. Additional file 2: Table S1. (Summary of data production and mapping results). Additional file 3: Table S2. (Variants (SNVs, Indels and CNVRs) identified in Pathan genome). Additional file 4: Table S3. (Functionally damaged novel nsSNVs). Additional file 5: Table S4. (Clinical relevance coding SNVs in Pathan whole genome). Additional file 6: Table S5. (Nonsynonymous SNVs in Pathan's genome). Additional file 7: Table S6. (Copy Number Variation Regions (CRVRs) in Pathan's genome). Additional file 8: Table S7. (Mitochondrial Haplotypes in Pathan's Genome). Additional file 9: Table S8. (Damaged nsSNVs and the drugs). Additional file 10: Table S9. (List of drugs (PharmGKB) in the Pathan's Genome).

## Abbreviations

PTN: Pathan
SNV: Single nucleotide variation
indels: Insertions and deletions
CDS: Coding DNA sequence
UTR: Untranslated regions
nsSNV: Nonsynonymous SNV
CNVR: Copy number variation region
MDS: Multidimensional scaling
SAIF: South Asian Indian Female
SJK: Seong-Jin Kim (First Korean Genome)

## References

[1] Lander ES, Linton LM, Birren B, Nusbaum C, Zody MC, Baldwin J (2001). Initial sequencing and analysis of the human genome. Nature.

[2] Veeramah KR, Hammer MF (2014). The impact of whole-genome sequencing on the reconstruction of human population history. Nat Rev Genet.

[3] Feero WG, Guttmacher AE (2014). Genomics, Personalized Medicine, and Pediatrics. Acad Pediatr.

[4] Sebastiani P, Timofeev N, Dworkis DA, Perls TT, Steinberg MH (2009). Genome‐wide association studies and the genetic dissection of complex traits. Am J Hematol.

[5] Wong LP, Ong RT, Poh WT, Liu X, Chen P, Li R (2013). Deep Whole-Genome Sequencing of 100 Southeast Asian Malays. Am J Hum Genet.

[6] Taus-Bolstad S (2008). Pakistan in Pictures.

[7] Cavalli-Sforza LL (2005). The human genome diversity project: past, present and future. Nat Rev Genet.

[8] Azim MK, Yang C, Yan Z, Choudhary MI, Khan A, Sun X (2013). Complete genome sequencing and variant analysis of a Pakistani individual. J Hum Genet.

[9] Sherry S, Ward M-H, Kholodov M, Baker J, Phan L, Smigielski E (2001). dbSNP: the NCBI database of genetic variation. Nucleic Acids Res.

[10] Wheeler DA, Srinivasan M, Egholm M, Shen Y, Chen L, McGuire A (2008). The complete genome of an individual by massively parallel DNA sequencing. Nature..

[11] Levy S, Sutton G, Ng PC, Feuk L, Halpern AL, Walenz BP (2007). The diploid genome sequence of an individual human. PLoS Biol..

[12] Gupta R, Ratan A, Rajesh C, Chen R, Kim HL, Burhans R (2012). Sequencing and analysis of a South Asian-Indian personal genome. BMC Genomics.

[13] Patowary A, Purkanti R, Singh M, Chauhan RK, Bhartiya D, Dwivedi OP (2012). Systematic analysis and functional annotation of variations in the genome of an Indian individual. Hum Mutat.

[14] Wang J, Wang W, Li R, Li Y, Tian G, Goodman L (2008). The diploid genome sequence of an Asian individual. Nature.

[15] Ahn SM, Kim TH, Lee S, Kim D, Ghang H, Kim DS (2009). The first Korean genome sequence and analysis: full genome sequencing for a socio-ethnic group. Genome Res.

[16] Dissanayake VH, Samarakoon PS, Scaria V, Patowary A, Sivasubbu S, Gokhale RS (2011). The Sri Lankan Personal Genome Project. Sri Lankan Pers Genome Proj.

[17] Dogan H, Can H, Otu HH (2014). Whole Genome Sequence of a Turkish Individual. PLoS One.

[18] Pushkarev D, Neff NF, Quake SR (2009). Single-molecule sequencing of an individual human genome. Nat Biotechnol.

[19] Tong P, Prendergast JG, Lohan AJ, Farrington SM, Cronin S, Friel N (2010). Sequencing and analysis of an Irish human genome. Genome Biol.

[20] Wang Y, Xu S, Liu Z, Lai C, Xie Z, Zhao C (2013). Meta-Analysis on the Association Between the TF Gene rs1049296 and AD. Can J Neurol Sci.

[21] Alvarez-Cubero MJ, Saiz M, Martinez-Gonzalez LJ, Alvarez JC, Lorente JA, Cozar JM (2013). Genetic analysis of the principal genes related to prostate cancer: a review. Urol Oncol.

[22] Aziz Z, Sana S, Saeed S, Akram M (2003). Institution based tumor registry from Punjab: five year data based analysis. J Pak Med Assoc.

[23] Bhurgri Y, Kayani N, Pervez S, Ahmed R, Tahir I, Afif M (2009). Incidence and Trends of Prostate Cancer in Karachi South. Asian Pac J Cancer Prev.

[24] Gueorguiev M, Lecoeur C, Meyre D, Benzinou M, Mein CA, Hinney A (2009). Association studies on ghrelin and ghrelin receptor gene polymorphisms with obesity. Obesity (Silver Spring).

[25] Bouchard L, Vohl M-C, Lebel S, Hould F-S, Marceau P, Bergeron J (2010). Contribution of genetic and metabolic syndrome to omental adipose tissue PAI-1 gene mRNA and plasma levels in obesity. Obes Surg.

[26] Galbete C, Toledo J, Martínez-González MÁ, Martínez JA, Guillén-Grima F, Marti A (2013). Lifestyle factors modify obesity risk linked to PPARG2 and FTO variants in an elderly population: a cross-sectional analysis in the SUN Project. Genes Nutr.

[27] Flegal KM, Carroll MD, Ogden CL, Curtin LR (2010). Prevalence and trends in obesity among US adults, 1999–2008. Jama.

[28] Kopelman PG, Caterson ID, Stock MJ, Dietz WH. Clinical obesity in adults and children: In Adults and Children. Blackwell Publishing. 2^nd^ Edition. 2005: 493.

[29] Streib L. World’s Fattest Countries. Forbes [http://www.forbes.com/2007/02/07/worlds-fattest-countries-forbeslife-cx_ls_0208worldfat.html]

[30] Tan J, Yang Y, Tang K, Sabeti PC, Jin L, Wang S (2013). The adaptive variant EDARV370A is associated with straight hair in East Asians. Hum Genet.

[31] Spichenok O, Budimlija ZM, Mitchell AA, Jenny A, Kovacevic L, Marjanovic D (2011). Prediction of eye and skin color in diverse populations using seven SNPs. Forensic Sci Int Genet.

[32] Sulem P, Gudbjartsson DF, Stacey SN, Helgason A, Rafnar T, Magnusson KP (2007). Genetic determinants of hair, eye and skin pigmentation in Europeans. Nat Genet.

[33] Yoshiura K, Kinoshita A, Ishida T, Ninokata A, Ishikawa T, Kaname T (2006). A SNP in the ABCC11 gene is the determinant of human earwax type. Nat Genet.

[34] Zheng T, Su CH, Zhao J, Zhang XJ, Zhang TY, Zhang LR (2013). Effects of CYP3A5 and CYP2D6 genetic polymorphism on the pharmacokinetics of diltiazem and its metabolites in Chinese subjects. Die Pharmazie.

[35] Li X, Lian FM, Guo D, Fan L, Tang J, Peng JB (2013). The rs1142345 in TPMT Affects the Therapeutic Effect of Traditional Hypoglycemic Herbs in Prediabetes. Evid Based Complement Alternat Med.

[36] Corrigan A, Lal R, Wickramasinghe S, Whelan S, Sanderson J, Marinaki A (2013). Testing for association between TPMT, COMT and NOX3 variants and the onset of ototoxicity in lung cancer patients treated with platinum chemotherapy [abstract]. Lung Cancer.

[37] Lindholm C (2004). Encyclopedia of Sex and Gender: Springer.

[38] Ayub Q, Tyler-Smith C (2009). Genetic variation in South Asia: assessing the influences of geography, language and ethnicity for understanding history and disease risk. Brief Funct Genomic Proteomic.

[39] Hewett M, Oliver DE, Rubin DL, Easton KL, Stuart JM, Altman RB (2002). PharmGKB: the pharmacogenetics knowledge base. Nucleic Acids Res.

[40] Wishart DS, Knox C, Guo AC, Cheng D, Shrivastava S, Tzur D (2008). DrugBank: a knowledgebase for drugs, drug actions and drug targets. Nucleic Acids Res.

[41] Ellingrod VL, Miller DD, Taylor SF, Moline J, Holman T, Kerr J (2008). Metabolic syndrome and insulin resistance in schizophrenia patients receiving antipsychotics genotyped for the methylenetetrahydrofolate reductase (< i > MTHFR</i>) 677C/T and 1298A/C variants. Schizophr Res.

[42] Jiang S, Hsu Y-H, Xu X, Xing H, Chen C, Niu T (2004). The C677T polymorphism of the methylenetetrahydrofolate reductase gene is associated with the level of decrease on diastolic blood pressure in essential hypertension patients treated by angiotensin-converting enzyme inhibitor. Thromb Res.

[43] Çetintaş VB, Erer OF, Kosova B, Özdemir I, Topçuoğlu N, Aktoğu S (2008). Determining the relation between N-acetyltransferase-2 acetylator phenotype and antituberculosis drug induced hepatitis by molecular biologic tests. Tuberk Toraks.

[44] Han K-M, Chang HS, Choi I-K, Ham B-J, Lee M-S (2013). CYP2D6 P34S Polymorphism and Outcomes of Escitalopram Treatment in Koreans with Major Depression. Psychiatry Invest.

[45] Andrews RM, Kubacka I, Chinnery PF, Lightowlers RN, Turnbull DM, Howell N (1999). Reanalysis and revision of the Cambridge reference sequence for human mitochondrial DNA. Nat Genet.

[46] Lee YS, Kim WY, Ji M, Kim JH, Bhak J (2009). MitoVariome: a variome database of human mitochondrial DNA. BMC Genomics.

[47] Loogväli EL, Roostalu U, Malyarchuk BA, Derenko MV, Kivisild T, Metspalu E (2004). Disuniting uniformity: a pied cladistic canvas of mtDNA haplogroup H in Eurasia. Mol Biol Evol.

[48] Mohyuddin A, Ayub Q, Qamar R, Zerjal T, Helgason A, Mehdi SQ (2001). Y-chromosomal STR haplotypes in Pakistani populations. Forensic Sci Int.

[49] Firasat S, Khaliq S, Mohyuddin A, Papaioannou M, Tyler-Smith C, Underhill PA (2006). Y-chromosomal evidence for a limited Greek contribution to the Pathan population of Pakistan. Eur J Hum Genet.

[50] Learn about Y-chromosome Haplogroup L. Genebase Tutorials. [http://64.40.115.136.van.ca.siteprotect.com/learning/article/13]

[51] Li H, Durbin R (2012). Inference of human population history from whole genome sequence of a single individual. Nature.

[52] Meyer M, Kircher M, Gansauge M-T, Li H, Racimo F, Mallick S (2012). A high-coverage genome sequence from an archaic Denisovan individual. Science.

[53] Speicher MR, Carter NP (2005). The new cytogenetics: blurring the boundaries with molecular biology. Nat Rev Genet.

[54] Li H, Durbin R (2009). Fast and accurate short read alignment with Burrows–Wheeler transform. Bioinformatics.

[55] Li H, Handsaker B, Wysoker A, Fennell T, Ruan J, Homer N (2009). The sequence alignment/map format and SAMtools. Bioinformatics.

[56] McKenna A, Hanna M, Banks E, Sivachenko A, Cibulskis K, Kernytsky A (2010). The Genome Analysis Toolkit: a MapReduce framework for analyzing next-generation DNA sequencing data. Genome Res.

[57] Miller CA, Hampton O, Coarfa C, Milosavljevic A (2011). ReadDepth: a parallel R package for detecting copy number alterations from short sequencing reads. PLoS One.

[58] Ng PC, Henikoff S (2003). SIFT: Predicting amino acid changes that affect protein function. Nucleic Acids Res.

[59] Jordan DM, Kiezun A, Baxter SM, Agarwala V, Green RC, Murray MF (2011). Development and validation of a computational method for assessment of missense variants in hypertrophic cardiomyopathy. Am J Hum Genet.

[60] Landrum MJ, Lee JM, Riley GR, Jang W, Rubinstein WS, Church DM (2013). ClinVar: public archive of relationships among sequence variation and human phenotype. Nucleic Acids Res.

[61] Wang K, Li M, Hakonarson H (2010). ANNOVAR: functional annotation of genetic variants from high-throughput sequencing data. Nucleic Acids Res.

[62] Salleh MZ, Teh LK, Lee LS, Ismet RI, Patowary A, Joshi K (2013). Systematic pharmacogenomics analysis of a Malay whole genome: proof of concept for personalized medicine. PLoS One.

[63] Pritchard JK, Stephens M, Donnelly P (2000). Inference of population structure using multilocus genotype data. Genetics.

[64] Prado-Martinez J, Sudmant PH, Kidd JM, Li H, Kelley JL, Lorente-Galdos B (2013). Great ape genetic diversity and population history. Nature.

