# Supplementary material for: Whole genome sequencing of an ethnic Pathan (Pakhtun) from the north-west of Pakistan
Source: BMC Genomics. 2015 Mar 12;16(1):172. doi: 10.1186/s12864-015-1290-1 (PMC4362645; doi:10.1186/s12864-015-1290-1)
Supplement: Additional file 1: Figure S1. — (Map of South Asia showing the Pathan/Pakhtun ethnic group in Pakistan and Afghanistan). Figure S2 (Comparative variant count of other reported individual genomes with Pathan genome. Figure S3 (Novel SNVs in personal genomes in thirteen different ethnic groups). Figure S4 (Comparing Pathan ethnic genome with other twelve diverse ethnic genomes from South Asia). Figure S5 (Cytogenetic analysis through GTG banding karyotype) and legends. [file 12864_2015_1290_MOESM1_ESM.doc]

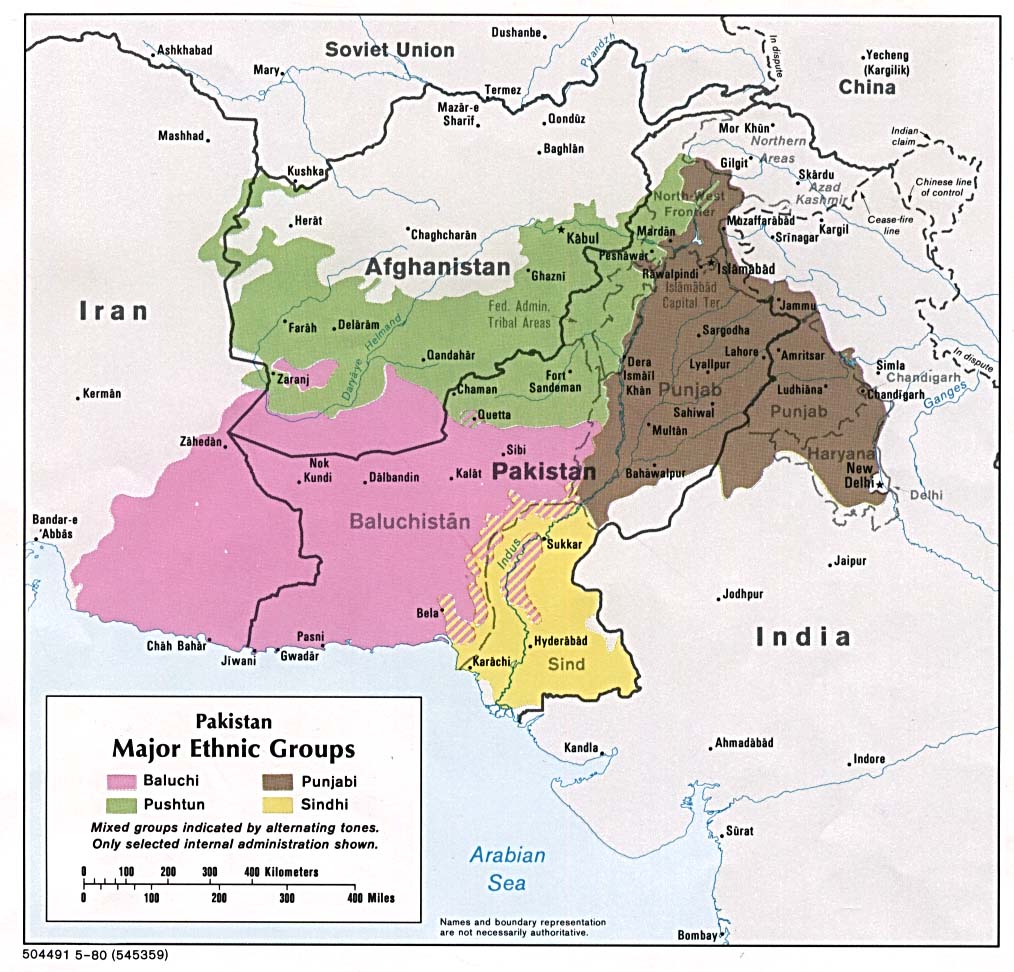


**Figure S1. Map of South Asia showing the Pathan / Pashtun ethnic group in Pakistan and Afghanistan.** Pathans are the largest ethnic group in Afghanistan and Pakistani provinces (Khyber Pakhtunkhwa) and Baluchistan of Pakistan. (http://amciv.wordpress.com/2009/05/07/pakistan/)


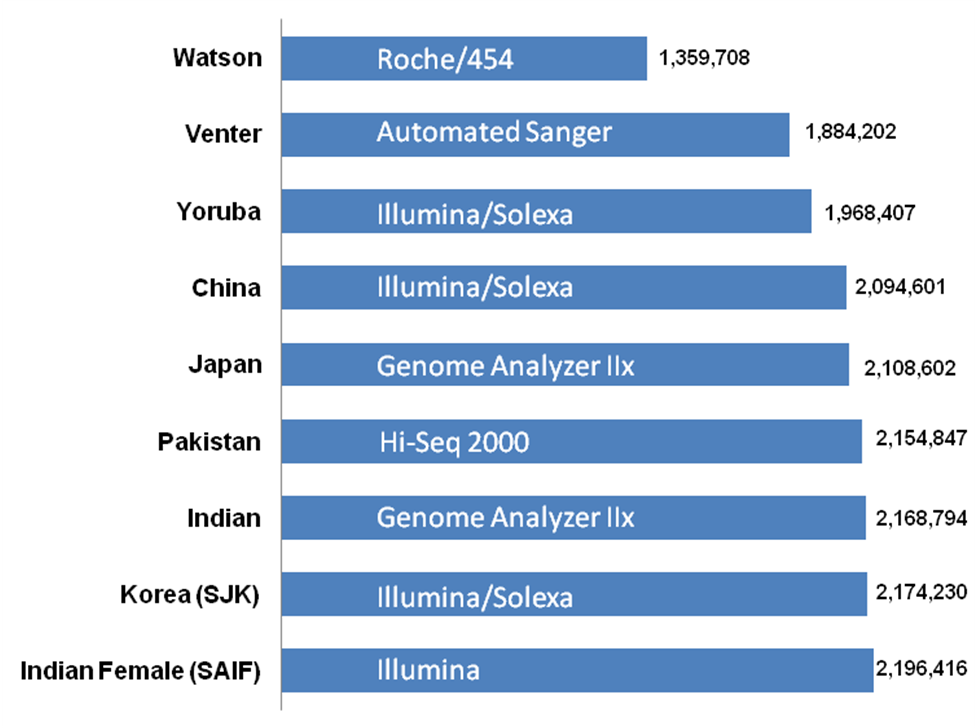


**Figure S2. Comparative variant count of other reported individual genomes with Pathan genome.** Graphical representation of comparative study of Pathans SNVs with other personal genomes reported previously.


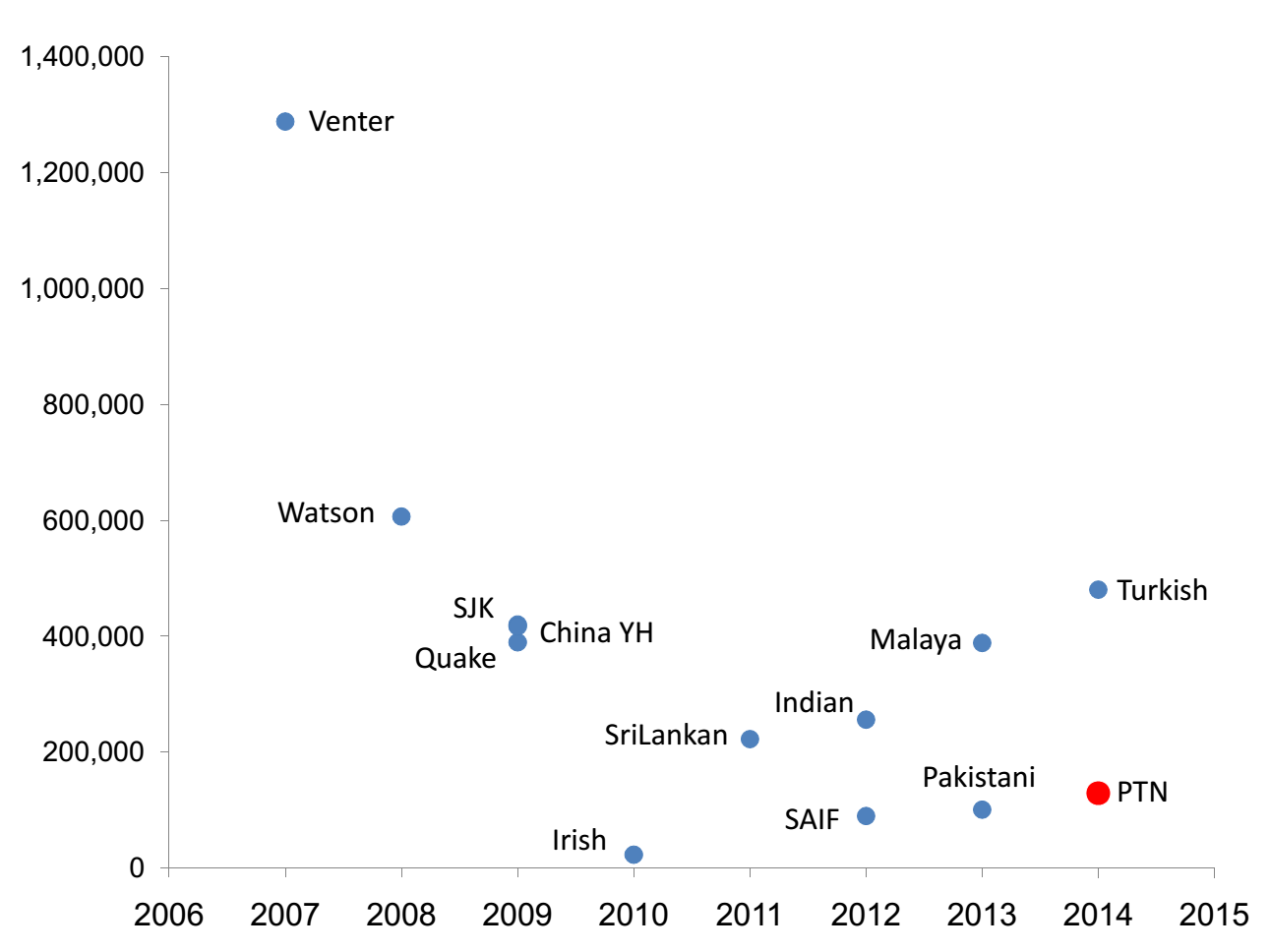


**Figure S3. Novel SNVs in personal genomes in thirteen different ethnic groups.** Scatter plot showing novel variants repoted in personal genomes. Data collected from literature.


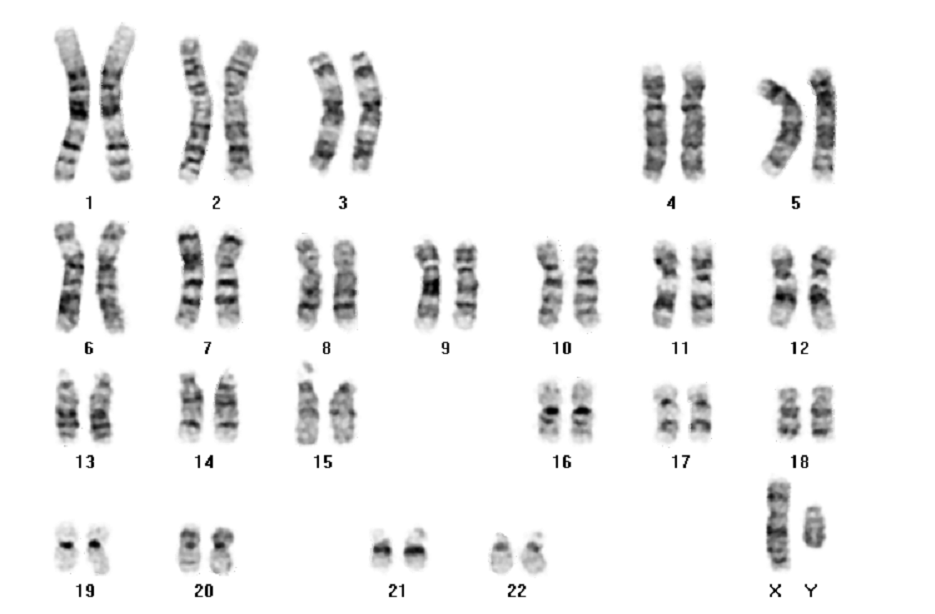


**Figure S4. Cytogenetic analysis through GTG banding karyotype.** Donor’s karyotype with 46 chromosomes (22 pairs of autosomes and X and Y sex chromosomes). Peripheral blood was used for cytogenetic studies in which chromosomes were treated with trypsin. The karyotype image shows normal chromosome number having no obvious structural aberrations.


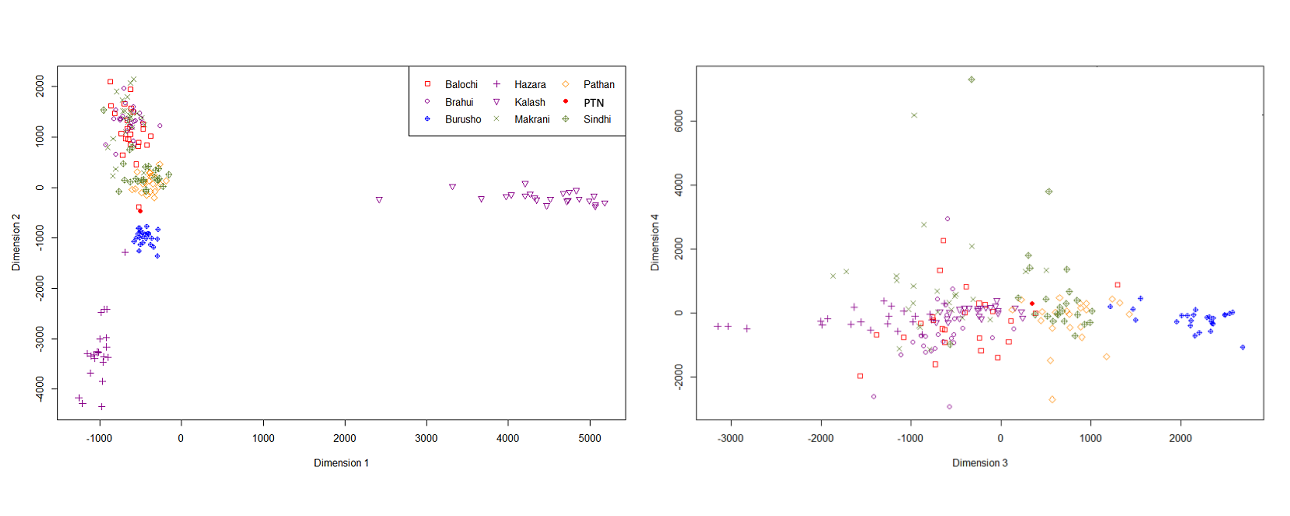


**Figure S5. Relationship of Pathan individual to other ethnic groups in South Asia.** Tweleve different groups from South Asia were compared with PTN. The analysis was based on 643,281 SNVs.
